# Isoflurane Conditioning Provides Protection against Subarachnoid Hemorrhage Induced Delayed Cerebral Ischemia through NF-kB Inhibition

**DOI:** 10.3390/biomedicines11041163

**Published:** 2023-04-12

**Authors:** Meizi Liu, Keshav Jayaraman, Jogender Mehla, Deepti Diwan, James W. Nelson, Ahmed E. Hussein, Ananth K. Vellimana, Yousef Abu-Amer, Gregory J. Zipfel, Umeshkumar Athiraman

**Affiliations:** 1 Department of Anesthesiology, Department of Neurosurgery, Washington University in St. Louis, St. Louis, MO 63110, USA; 2 Department of Orthopedic Surgery and Cell Biology & Physiology, Shriners Hospital for Children, Washington University School of Medicine, St. Louis, MO 63110, USA

**Keywords:** isoflurane, aneurysmal subarachnoid hemorrhage, delayed cerebral ischemia, NF-kB, neuroprotection

## Abstract

Delayed cerebral ischemia (DCI) is the largest treatable cause of poor outcome after aneurysmal subarachnoid hemorrhage (SAH). Nuclear Factor Kappa-light-chain-enhancer of Activated B cells (NF-kB), a transcription factor known to function as a pivotal mediator of inflammation, is upregulated in SAH and is pathologically associated with vasospasm. We previously showed that a brief exposure to isoflurane, an inhalational anesthetic, provided multifaceted protection against DCI after SAH. The aim of our current study is to investigate the role of NF-kB in isoflurane-conditioning-induced neurovascular protection against SAH-induced DCI. Twelve-week-old wild type male mice (C57BL/6) were divided into five groups: sham, SAH, SAH + Pyrrolidine dithiocarbamate (PDTC, a selective NF-kB inhibitor), SAH + isoflurane conditioning, and SAH + PDTC with isoflurane conditioning. Experimental SAH was performed via endovascular perforation. Anesthetic conditioning was performed with isoflurane 2% for 1 h, 1 h after SAH. Three doses of PDTC (100 mg/kg) were injected intraperitoneally. NF-kB and microglial activation and the cellular source of NF-kB after SAH were assessed by immunofluorescence staining. Vasospasm, microvessel thrombosis, and neuroscore were assessed. NF-kB was activated after SAH; it was attenuated by isoflurane conditioning. Microglia was activated and found to be a major source of NF-kB expression after SAH. Isoflurane conditioning attenuated microglial activation and NF-kB expression in microglia after SAH. Isoflurane conditioning and PDTC individually attenuated large artery vasospasm and microvessel thrombosis, leading to improved neurological deficits after SAH. The addition of isoflurane to the PDTC group did not provide any additional DCI protection. These data indicate isoflurane-conditioning-induced DCI protection after SAH is mediated, at least in part, via downregulating the NF-kB pathway.

## 1. Introduction

Aneurysmal subarachnoid hemorrhage (SAH) can cause catastrophic brain injury and often affects younger patients. Morbidity and mortality (~30%) remain unacceptably high after SAH and a significant proportion of survivors (50%) have long-term cognitive deficits that prevent these patients from returning to their regular activities [1,2]. Delayed cerebral ischemia (DCI) occurs in ~30% of patients and develops 4–12 days after the initial bleed [2]. Though the major contributor for DCI is cerebral vasospasm, we now understand that additional processes, which affect microcirculation, play causal roles in DCI [3,4]. Autoregulatory dysfunction and microvessel thrombosis are both critical contributors [3,4]. We draw two key conclusions from this improved understanding of DCI: (1) Several previous therapies have failed so far, probably due to focusing on one particular component of DCI, rather than aiming at multiple factors; and (2) Prospective therapeutic strategies should focus both on the micro- and macrovascular components of DCI. This principle—new SAH therapies need to target multiple DCI processes—provides a strong rationale why conditioning-based therapy, which engages powerful pleiotropic endogenous protective cascades, has high potential for improving outcomes for SAH patients.

Conditioning is the concept whereby the brain’s inherent resistance to injury can be enhanced by exposure to a sub-lethal injurious stimulus. The adaptive responses induced by conditioning involve molecular sensors and transducers, transcription factors, genes, and effectors that ultimately produce a cerebroprotective phenotype [5]. Neurons were considered to be the primary target of this conditioning response, but now glial cells and vasculature have also been shown to play a significant role [5,6,7]. We and others have previously demonstrated the neuroprotective role of volatile anesthetics in DCI after SAH in several preclinical and clinical studies [8,9,10,11,12]. Specifically, we showed that conditioning with isoflurane (a volatile anesthetic) provided strong protection against multiple components of DCI, leading to improved short-term neurological deficits in a murine model of SAH [8,10]. Identifying the molecular mechanisms by which isoflurane conditioning provides DCI protection is critical for enhancing the translational potential of conditioning-based treatment for SAH patients. Specifically, understanding the molecular pathways by which isoflurane provides DCI protection will identify new molecular targets for drug development.

Accumulating evidence implicates inflammation as a key driver in DCI after SAH; however, the drivers of inflammation and the mechanisms leading to injury remain incompletely understood [13,14]. Nuclear Factor Kappa-light-chain-enhancer of Activated B cells (NF-kB) is a key transcriptional factor involved in inflammation and is shown to be activated after SAH [15,16,17]. NF-kB is a protein complex sequestered in the cytoplasm by a regulatory complex called inhibitors of kB (IkB) until it is activated by another protein complex called IkB kinase (IKK) [18,19]. In the canonical pathway, activation of IKK (specifically IKK2) results in the phosphorylation of IkB (specifically IkBα), leading to the nuclear translocation of NF-kB subunits (p65, p50) and the transcription of proinflammatory genes [18,19]. Although the impact of NF-kB has been described in several neurological disorders, including Alzheimer’s disease, Parkinson’s disease, and amyotrophic lateral sclerosis [20], its role in SAH-induced DCI and neurologic outcome has not been explored in detail. The first hint of a potential link between NF-kB and SAH-induced brain injury came when Zhou and colleagues [15,16] noted, in a rabbit model of SAH, that NF-kB DNA activity is increased after SAH and that administration of the NF-kB-specific inhibitor, pyrrolidine dithiocarbamate (PDTC), reduces large artery vasospasm and neuronal death. Though preliminary evidence suggests pharmacologic inhibition of NF-kB attenuates vasospasm after SAH [15], its impact on non-vasospasm elements of DCI, such as microvessel thrombi, autoregulatory dysfunction and neurological outcome (short and long-term), remains to be determined. In addition, the cellular source of NF-kB in SAH has not been established. This is important as NF-kB is also involved in critical physiological roles in the brain; as such, complete inhibition of NF-kB may lead to detrimental effects. Therefore, identifying the specific cellular source of NF-kB that contributes to its overproduction after SAH is important for the development of targeted drug therapeutics for this devastating condition. In addition, the role of NF-kB in the DCI and neurological protection afforded by isoflurane conditioning in SAH has not been explored. The aim of our current study is to examine the role of NF-kB in isoflurane-conditioning-induced DCI protection.

## 2. Materials and Methods

### 2.1. Experiment Design

**Figure 1:**
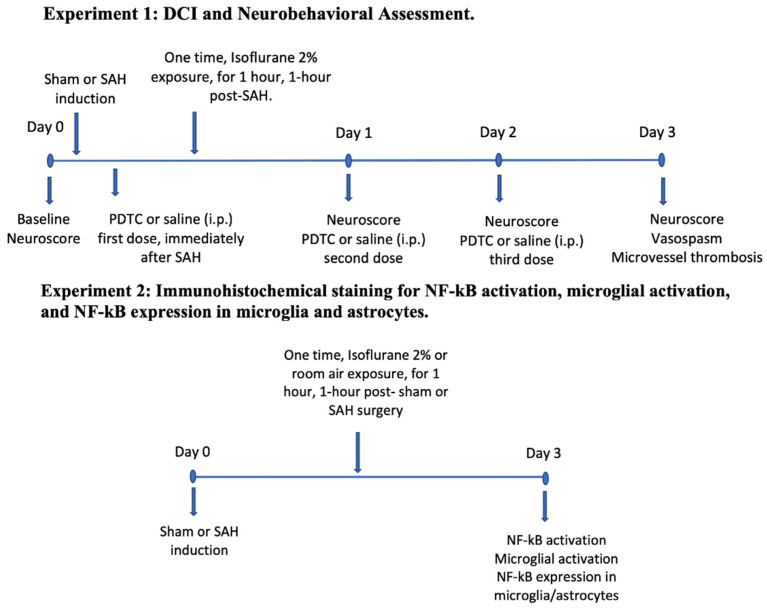
Experimental design of the study. SAH—subarachnoid hemorrhage. DCI—delayed cerebral ischemia. PDTC—Pyrrolidine dithiocarbamate. NF-kB—Nuclear factor kappa-light-chain-enhancer of activated B cells. i.p.—intraperitoneal.

All experiments conducted in the study were approved by the Animal Ethical Board Committee at Washington University in Saint Louis, USA (Protocol no. 20180080, Approval date 22 July 2019). All experiments were performed in a blinded and randomized method. Twelve-week-old C57BL/6J wild type male mice were obtained from Jackson Laboratories (Bar Harbor, ME, USA) for the experiments. Animals were kept in a 12 h dark-light cycle in our approved animal facility with free access to water and a standard mouse diet. Experimental mice were separated into the following groups for DCI and neurobehavioral assessment: sham (*n* = 17), SAH (*n* = 13), SAH + PDTC (*n* = 17), SAH + isoflurane conditioning (*n* = 11), and SAH + isoflurane conditioning + PDTC (*n* = 17). Immunohistochemistry experiments were conducted to assess NF-kB and microglial activation, as well as to identify the major cellular source of NF-kB after SAH. All animals underwent SAH induction and isoflurane conditioning during the light phase of the 12 h dark-light cycle. DCI, neuroscore, and all immunohistochemical experiments were assessed during the light phase of the 12 h dark-light cycle. Eighty-five mice were used in the experiments. Six mice died in the SAH groups, and the cerebral vessels were not seen clearly in four mice due to inadequate staining. Therefore, a total of 10 mice were excluded from the final analysis. The overall experimental design of the study is represented in Figure 1.

### 2.2. Endovascular Perforation SAH Mouse Model

As per our previously published methods, SAH was created via endovascular perforation [8,10]. During a brief period of isoflurane anesthesia with medical air as the carrier gas, a midline incision was made in the neck in the prone position to expose the external carotid artery (ECA). After the exposure, a 5-0 nylon suture was passed through the ECA until it reached the internal carotid artery (ICA) bifurcation. In the SAH groups, the suture was advanced further to cause perforation; the suture was removed without causing perforation in the sham group. Isoflurane duration during surgery was brief, approximately 10–15 min in sham and 15–20 min in SAH groups. Animals that died after surgery, those where blood was not present in the SAH group, or if blood was present in the sham group were excluded from the analysis. The rest of the sham and SAH animals were included in the analysis. 

### 2.3. Anesthetic Conditioning

Anesthetic conditioning was established by exposing mice to isoflurane after subarachnoid hemorrhage induction; this is referred to as isoflurane post-conditioning (postC). Isoflurane 2% with medical air was administered to mice for one hour, beginning one-hour post SAH. A medium sized surgi vet anesthetic induction chamber (Smiths Medical, Dublin, OH, USA) was used for isoflurane conditioning. Isoflurane concentration in the chamber was measured using a gas analyzer (Datex Ohmeda, Capnomac Ultima, Louisville, KY, USA). Normothermia at 37 degrees Celsius was maintained using a homeothermic blanket. Since 2% isoflurane for one hour has been used safely in several studies [8,10,11,12], we used the same percentage and duration of isoflurane exposure in our experiments.

### 2.4. Pyrrolidine Dithiocarbamate (PDTC) Treatment

Three doses of PDTC (100 mg/kg) diluted with saline were injected intraperitoneally; one dose was injected immediately after SAH induction, and two doses were repeated on day 1 and 2 after SAH. PDTC was freshly diluted for each day’s injections. Other groups received the same volume of saline intraperitoneally.

### 2.5. Neurobehavioral Assessment

Neurological function was assessed per our published methods [8,10]. Briefly, neuroscore was measured at baseline before SAH and repeated for the next three days until the animals were sacrificed. The neurological function was graded based on a motor score (0 to 12) and a sensory score (4 to 12). The motor score was assessed by spontaneous activity, symmetry of limb movements, climbing, and balance and coordination. The sensory score was assessed by body proprioception, vibrissa, visual, and tactile responses.

### 2.6. Vasospasm Assessment

According to our published literature [8,10], mice were anesthetized with isoflurane and transcardiac perfusion with ROX SE (5-(and-6)-Carboxy-X-rhodamine, succinimidyl ester) was performed 3 days after SAH surgery. The left (ipsilateral) middle cerebral artery (MCA) vessel was visualized using a fluorescent microscope (CoolSNAP EZ, Photometrics, Tucson, AZ, USA) and MetaMorph^®^ software (Universal Imaging, West Chester, PA, USA) to measure vasospasm. The narrowest width in the first 1 mm of the MCA was taken as the measure of vasospasm.

### 2.7. Microvessel Thrombosis Assessment

As per our published methods [10], ipsilateral (left) cortical microvessel thrombosis was evaluated for fibrinogen by immunofluorescence staining seventy-two hours post SAH. Briefly, free floating, fixed brain sections with 50-μm thickness were incubated overnight at 4 °C with primary rabbit anti-fibrinogen antibody (1:3000, Abcam, Cambridge, MA, USA), followed by overnight incubation at 4 °C with donkey anti-rabbit secondary antibody (1:2000, Invitrogen Waltham, MA, USA). Brain sections were then analyzed for fibrinogen using a NanoZoomer 2.0-HT digital slide scanner (Hamamatsu Corporation, Bridgewater, NJ, USA), followed by estimation of microvessel thrombosis density using Image J (v.1.53t).

### 2.8. Immunostaining

Mice underwent transcardiac perfusion on day 3 after SAH and brains were stored and fixed in 4% paraformaldehyde. Frozen brain samples were sectioned into 30 μm coronal sections using microtome. Brain slices were then incubated with a blocking buffer (0.4% Triton X-100 TBS-T with 5% donkey serum) for 2 h at room temperature and then incubated with primary antibodies (rabbit anti-NF-kB, #8242, Cell Signaling, Danvers, MA, USA {1:100}; Goat Anti-Iba-1, ab5076, Abcam, MA, USA {1:500}; Goat Anti-GFAP, A-31553, ThermoFisher, Waltham, MA, USA {1:200}; Rat anti-CD68, MCA1957, BIO-RAD, CA, USA {1:500}), followed by incubation at room temperature with secondary antibodies (Donkey anti-Rabbit Alexa Flour 488, A32790, ThermoFisher {1:1000}; Donkey anti-Goat Alexa Fluor 647, A-21447, ThermoFisher {1:1000}; Donkey anti-Rat Alexa Fluor 488, A-21208, ThermoFisher {1:1000}) in TBS-T for 45 min. After rinsing, the brain sections were stained with DAPI and mounted on the slide. The images were taken using Nikon Confocal microscopy. The fluorescent signaling was analyzed using Image J.

### 2.9. Statistical Analysis

Data are represented as Mean ± Standard error of mean. Normality of the data was assessed using the Shapiro–Wilk test. Neurobehavioral measurement was analyzed using two-way repeated measures ANOVA followed by Student–Newman–Keuls multiple comparison test. Large artery vasospasm, microvessel thrombosis, and immunofluorescence staining for NF-kB and microglial activation were analyzed using one-way ANOVA followed by Student–Newman–Keuls multiple comparison test. NF-kB colocalization with microglia and astrocytes were analyzed by grouped two-way ANOVA followed by Student–Newman–Keuls multiple comparison test. *p* < 0.05 was considered to be statistically significant. 

## 3. Results

### 3.1. Isoflurane Conditioning Attenuates NF-kB Activation after SAH

**Figure 2:**
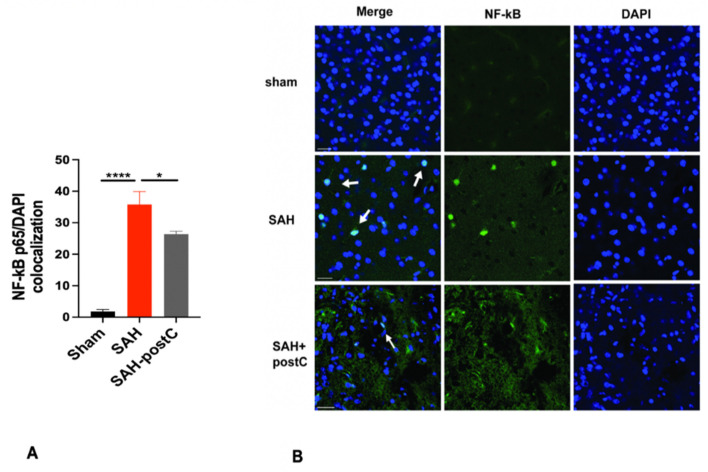
(**A,B**): Isoflurane conditioning attenuates NF-kB activation after SAH in an endovascular perforation model: Wild type male mice underwent SAH or sham surgery followed, 1 h later, by exposure to 2% isoflurane (PostC) or room air for 1 h. NF-kB/DAPI colocalization assessment was performed by immunofluorescence staining in brain around the vascular perforation area. Data represent mean ± SEM. (**A**) * *p* < 0.05 Sham vs. SAH, * *p* < 0.05, SAH vs. SAH-postC by ANOVA with Newman–Keuls multiple comparisons test. (*n* = 5). (**B**) Representative immunofluorescence staining images for NF-kB/DAPI colocalization. Arrow mark shows the NF-kB/DAPI colocalization. Scale bar = 50 μm. SAH—subarachnoid hemorrhage. postC—isoflurane post conditioning. (* *p* < 0.05, **** *p* < 0.0001).

NF-kB activation was examined on day 3 by investigating its colocalization with DAPI. NF-kB was significantly activated after SAH when compared to the sham group (*p* < 0.05, Figure 2A,B) and isoflurane conditioning significantly reduced the nuclear presence of NF-kB (a critical step for activation) (*p* < 0.05, Figure 2A,B).

### 3.2. Isoflurane Conditioning Reduces Microglial Activation and NF-kB Expression in Microglia after SAH

**Figure 3:**
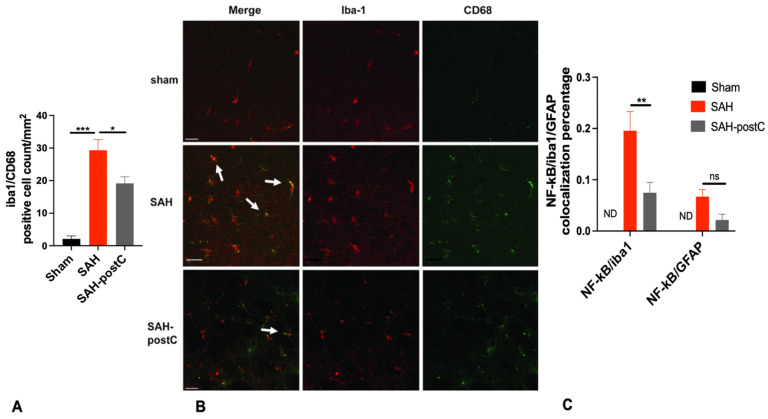

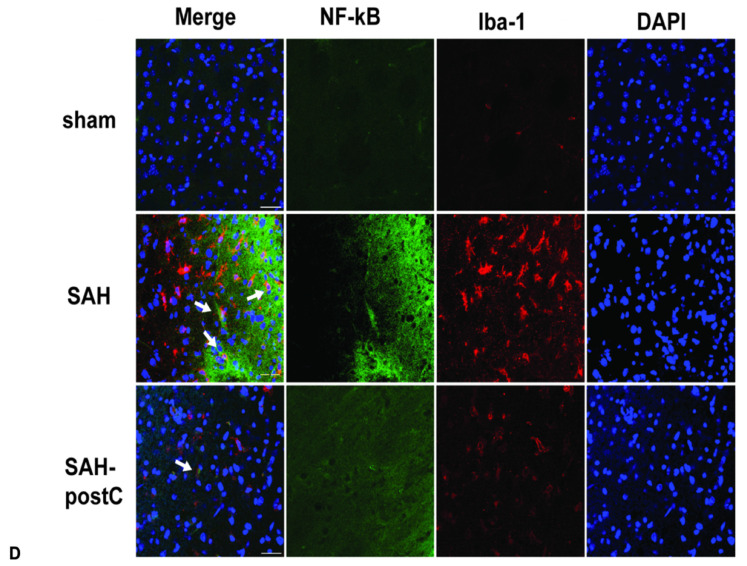

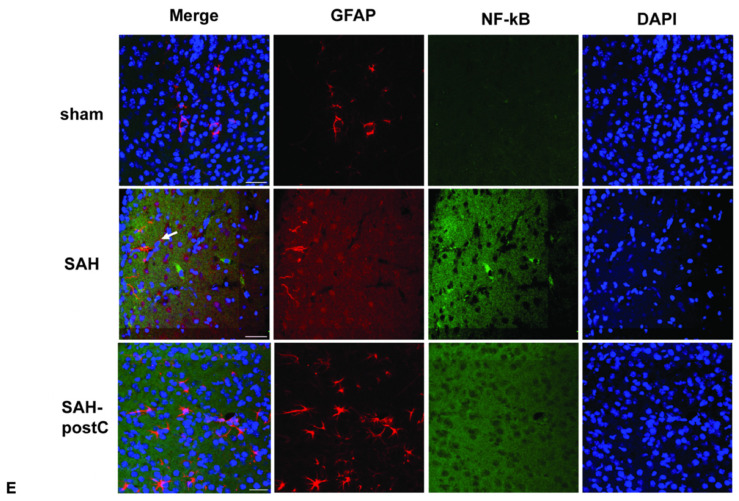
(**A**–**E**): Isoflurane conditioning attenuates microglial activation and NF-kB expression in microglia after SAH in an endovascular perforation model: Wild type male mice underwent SAH or sham surgery followed, 1 h later, by exposure to 2% isoflurane (PostC) or room air for 1 h. (**A**,**B**) Microglial activation (iba1/CD68) and (**C**–**E**) NF-kB colocalization with microglia/astrocytes (NF-kB/DAPI/iba-1, NF-kB/DAPI/GFAP) was assessed by immunofluorescence staining in the ipsilateral brain (left) around the vessel perforation area, 3 days after SAH. Data represent mean ± SEM. (**A**) * *p* < 0.05 Sham vs. SAH, * *p* < 0.05 SAH vs. SAH-postC by ANOVA with Newman–Keuls multiple comparisons test, (**C**) * *p* < 0.05 SAH vs. SAH-postC, ND-not detected in sham group, nonsignificant (ns) by grouped two-way ANOVA with Newman–Keuls multiple comparisons test. (*n* = 3–6) (**B**,**D**,**E**) Representative immunofluorescence staining images for microglial activation (**B**), NF-kB/DAPI/iba-1 (**D**), NF-kB/DAPI/GFAP (**E**) colocalization. Scale bar = 50 μm. SAH—subarachnoid hemorrhage. postC—isoflurane post conditioning. iba1—ionized calcium binding adaptor molecule. GFAP—Glial fibrillary acidic protein. (* *p* < 0.05, ** *p* < 0.01, *** *p* < 0.001).

SAH causes strong activation of microglia, which was attenuated by isoflurane conditioning (*p* < 0.05, Figure 3A,B)). We also show that microglia are the major source of increased NF-kB expression after SAH, and that isoflurane conditioning strongly attenuates this pathologic molecular response (*p* < 0.05, Figure 3C,D).

### 3.3. Isoflurane Conditioning and PDTC Administration Independently Affords Strong Protection against SAH-Induced Vasospasm and Neurobehavioral Deficits—Combining Isoflurane Conditioning with PDTC Did Not Provide Additional Protection

**Figure 4:**
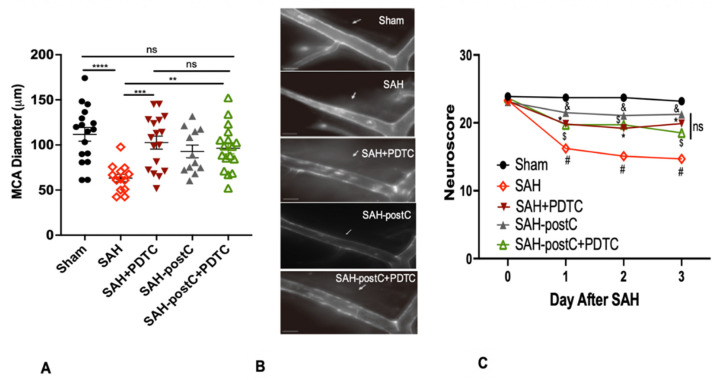
(**A**–**C**): Isoflurane conditioning and PDTC protects against SAH-induced vasospasm and neurological deficits in an endovascular perforation model: Wild type male mice were subjected to sham surgery or endovascular perforation SAH and treated with vehicle (saline) or three doses of PDTC 100 mg/kg with one PDTC group exposed to 2% isoflurane for one hour, one hour after SAH. A separate cohort of SAH mice was exposed only to 2% isoflurane for one hour, beginning one hour after SAH. Vasospasm was assessed on Day 3 (**A**). Neuroscore was assessed baseline and daily for 3 days (**C**). Data represent mean ± SEM. (**A**) * *p* < 0.05 Sham vs. SAH, * *p* < 0.05 SAH vs. SAH + PDTC, SAH-postC, and SAH-postC + PDTC, (ns) * *p* > 0.05 SAH + PDTC vs. SAH-postC vs. SAH-postC + PDTC. (**C**) # *p* < 0.05 Sham vs. SAH, * *p* < 0.05 SAH vs. SAH + PDTC, and *p* < 0.05 SAH vs. SAH-postC, $ *p* < 0.05 SAH vs. SAH-postC + PDTC. (ns) *p* > 0.05 SAH + PDTC vs. SAH-postC vs. SAH-postC + PDTC, by ANOVA and two-way repeated measures ANOVA with Newman–Keuls multiple comparisons test. (**B**) Representative images for vasospasm. The arrow mark points to ipsilateral left middle cerebral artery. ns—nonsignificant. SAH—subarachnoid hemorrhage. postC—isoflurane post conditioning. PDTC—Pyrrolidine dithiocarbamate. (** *p* < 0.01, *** *p* < 0.001, **** *p* < 0.0001).

Of the 85 mice used in the experiments, six mice died after SAH; none died in the sham group. The MCA vessel was unable to be visualized in four mice. Mice in the SAH group were noticed to have SAH; none in the sham group was noticed to have SAH. Mice subjected to SAH had significant vasospasm and neurologic deficits; these were attenuated by the administration of isoflurane or PDTC (*p* < 0.05, Figure 4). Coadministration of isoflurane and PDTC did not have any additive protective effect when compared to the isoflurane or PDTC groups alone (*p* > 0.05, Figure 4).

### 3.4. Isoflurane Conditioning and PDTC Administration Independently Provides Strong Protection against Microvessel Thrombosis Induced by SAH—Combining Isoflurane Conditioning with PDTC Did Not Provide Additional Protection

**Figure 5:**
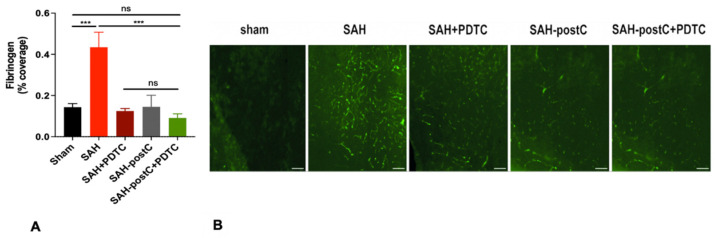
(**A**,**B**): PDTC protects against SAH-induced microvessel thrombosis in an endovascular perforation model: Wild type male mice were subjected to sham surgery or endovascular perforation SAH and treated with vehicle (saline) or three doses of PDTC 100 mg/kg with one PDTC group exposed to 2% isoflurane for one hour, one hour after SAH. A separate cohort of SAH mice was exposed only to 2% isoflurane for one hour, beginning one hour after SAH. Microvessel thrombosis was assessed on Day 3 ((**A**), *n* = 2–5). Data represent mean ± SEM. (**A**) * *p* < 0.05 Sham vs. SAH, * *p* < 0.05 SAH vs. SAH + PDTC, SAH-postC, and SAH-postC + PDTC, (ns) *p* > 0.05 SAH + PDTC vs. SAH-postC vs. SAH-postC + PDTC, by ANOVA with Newman–Keuls multiple comparison test. (**B**) Representative images for microvessel thrombosis. Scale corresponds to 100 μM. ns—nonsignificant. SAH—subarachnoid hemorrhage. postC—isoflurane post conditioning. PDTC—Pyrrolidine dithiocarbamate. (*** *p* < 0.001).

Next, we investigated if NF-kB inhibition by PDTC reduces microvessel thrombosis and if the addition of isoflurane to PDTC has any additional effect. As expected, significant microvessel thrombosis was noticed in the SAH group when compared to the sham group (*p* < 0.05, Figure 5); administration of isoflurane or PDTC reduced microvessel thrombosis (*p* < 0.05, Figure 5), but isoflurane coadministration with PDTC did not have any additive effect compared to the isoflurane or PDTC groups alone (*p* > 0.05, Figure 5).

## 4. Discussion

The main findings in our study are: (1) NF-kB is activated after SAH and isoflurane conditioning attenuated the NF-kB activation; (2) Microglia is activated and primarily expressed NF-kB after SAH; isoflurane conditioning attenuated microglial activation and reduced NF-kB expression in microglia after SAH; (3) PDTC, a selective NF-kB inhibitor, or isoflurane conditioning individually offers strong protection against SAH-induced large artery vasospasm and improves neurobehavioral deficits; supplementing isoflurane conditioning to PDTC did not provide any additional benefit; and (4) Isoflurane or PDTC also protected against a microvascular component of DCI, namely microvessel thrombosis, and complementing isoflurane conditioning to PDTC did not afford any additional protection. These findings indicate that (1) isoflurane-conditioning-induced DCI protection against SAH appears to be mediated through downregulating NF-kB, and (2) PDTC is a promising new therapeutic for DCI protection after SAH. 

Recent evidence suggests that neuroinflammation is a critical driver in the progression of secondary brain injury after SAH, leading to poor neurobehavioral outcomes [13,14]. NF-kB, a transcription factor, is a key regulator of inflammation and immune functions. Dysregulated NF-kB signaling can result in the overactivation of proinflammatory genes, such as cytokines, chemokines and inflammasomes, resulting in tissue damage and the development of acute and chronic inflammatory diseases [18,19]. Multiple studies have shown that NF-kB is critically involved in the pathogenesis of several systemic disorders, including rheumatoid arthritis, inflammatory bowel disease, systemic lupus erythematosus, chronic obstructive pulmonary disease, asthma, and others [20]. It is also shown that NF-kB is activated in several central nervous system disorders, including Alzheimer’s disease, Huntington’s disease, Parkinson’s disease, amyotrophic multiple sclerosis, traumatic brain injury, spinal cord injury, and ischemic brain injury [21,22,23]. 

Interestingly, a study by Zhou et al. showed that NF-kB is activated after SAH in isolated cerebrovascular smooth muscle cells treated with hemolysate and PDTC suppressed NF-kB activation [15]. They also demonstrated the occurrence of severe vasospasm in a double injection cisterna magna SAH model in rabbits; the administration of PDTC significantly reduced vasospasm [15]. A subsequent study by the same group, using a similar SAH model, demonstrated that NF-kB is activated after SAH and was primarily localized in the neurons [16]. The activated NF-kB in the neurons resulted in the expression of other pro inflammatory genes, such as tumor necrosis factor-alpha, Interleukin -1 beta, and intercellular adhesion molecule (ICAM)-1, leading to delayed brain injury. They also showed that administration of PDTC attenuated NF-kB activation, expression of the pro inflammatory genes, and ameliorated cell death, suggesting that PDTC could possibly mitigate delayed brain injury after SAH [16]. An in vitro study published by Cheng et al. demonstrated that cerebrovascular smooth muscle cells stimulated via oxyhemoglobin expresses endothelin-1(ET-1) (a potent vasoconstrictor), and that its upregulation was activated by NF-kB [17]. Authors also showed that PDTC downregulated ET-1 expression and decreased the contractile response of the arterial rings exposed to oxyhemoglobin, speculating that inhibition of NF-kB pathway could be beneficial in the treatment of cerebral vasospasm after SAH [17]. 

Our data show that NF-kB is activated after SAH and that administration of isoflurane significantly attenuated NF-kB activation. These results match with previous reports demonstrating that isoflurane conditioning provided neuroprotection in ischemic brain injuries by attenuating neuroinflammation via inhibiting NF-kB [24,25,26]. It is possible that isoflurane prevents the translocation of NF-kB from the cytoplasm to nucleus, a key event necessary for NF-kB to produce the proinflammatory genes required for inflammation. 

Next, we sought to examine the cellular source of the aberrant NF-kB in SAH. Our experiments show that microglia are activated after SAH and that they mediate increased NF-kB expression in brain. Interestingly, microglial activation has been shown to occur after SAH and that pharmacological depletion of microglia attenuated vasospasm; this suggests that microglia are important contributors to DCI [27,28]. We also show that isoflurane conditioning attenuates both microglial activation and NF-kB expression in microglia after SAH. Whether such attenuation plays a role in isoflurane-induced protection against DCI and neurological deficits after SAH remains to be determined. 

We also show that intraperitoneal administration of PDTC, a NF-kB inhibitor, provided robust protection against multiple elements of SAH-induced DCI, including large artery vasospasm, microvessel thrombosis, and neurological deficits. Though one previous study has shown that PDTC provides protection against vasospasm [15], other elements of DCI, such as microvessel thrombosis and the neurological outcome, were not examined in that study. Our study builds upon this preliminary study by showing that PDTC protects against multiple elements of DCI, thereby leading to improved neurologic outcomes. 

Next, we wanted to examine if administration of PDTC combined with isoflurane conditioning has any additional beneficial effect on DCI and neurologic outcomes after SAH. Interestingly, we noticed that co-administration of PDTC with isoflurane conditioning did not provide any additive protective effect against DCI or the neurologic outcomes. This finding suggests that the DCI protection afforded by isoflurane conditioning is mediated via inhibiting NF-kB.

## 5. Limitations

There are a few limitations in our study: (1) although the endovascular perforation model closely mimics SAH in humans, our results must be confirmed with other animal models; (2) further studies to determine the pathophysiological role of downstream effectors of NF-kB in SAH-induced brain injury and the impact of isoflurane conditioning are required; (3) additional studies examining the impact of conditional deletion of NF-kB in microglia on SAH-induced DCI and neurologic deficits are necessary; and (4) the effects of isoflurane conditioning or PDTC on long-term neurobehavioral outcomes after SAH were not examined. This is essential when considering translational studies in patients. 

## 6. Conclusions

Overall, we conclude that isoflurane-conditioning-induced DCI protection is, at least in part, facilitated through NF-kB inhibition and that PDTC is a potential therapeutic target to prevent secondary brain injury after SAH and to improve neurologic outcomes. 

## Data Availability

All data in the study are available by a reasonable request to the corresponding author, Umeshkumar Athiraman (uathira@wustl.edu).

## Abbreviations

SAH	Subarachnoid hemorrhage.
DCI	Delayed Cerebral Ischemia.
NF-kB	Nuclear Factor Kappa-light-chain-enhancer of Activated B cells.
PDTC	Pyrrolidine dithiocarbamate.
DAPI	4′,6-diamidino-2-phenylindole.
MCA	middle cerebral artery.
ICA	Internal carotid artery.
ECA	External Carotid artery.
ROX-SE	5-(and-6)-Carboxy-X-rhodamine, succinimidyl ester.
PBS	Phosphate buffered saline.
TBS-T	Tris Buffered Saline + Tween.
ANOVA	Analysis of Variance.
